# Low Reynolds Number Swimming Near Interfaces in Multi-Fluid Media

**DOI:** 10.3390/app11199109

**Published:** 2021-09-30

**Authors:** Avriel Cartwright, Jian Du

**Affiliations:** Department of Mathematical Sciences, Florida Institute of Technology, Melbourne, FL 32901, USA

**Keywords:** locomotion, two-fluid mixture, immersed boundary method, free interface

## Abstract

Microorganisms often swim within heterogeneous fluid media composed of multiple materials with very different properties. The swimming speed is greatly affected by the composition and rheology of the fluidic environment. In addition, biological locomotions are also strongly influenced by the presence of phase boundaries and free interfaces, across which physical properties of the fluid media may vary significantly. Using a two-fluid immersed boundary method, we investigate the classical Taylor’s swimming sheet problem near interfaces within multi-fluid media. The accuracy of the methodology is illustrated through comparisons with analytical solutions. Our simulation results indicate that the interface dynamics and phase separation in the multi-fluid mixture are closely coupled with the movement of the swimmer. Depending on the interface location, the frictional coefficient, and the multi-fluid composition, the swimmer can move faster or slower than that in a single phase fluid.

## 1. Introduction

Recently, there has been a surge of research interest in the locomotion of microorganisms through their fluidic environment. Examples include the swimming of the ulcer-causing Helicobacter pylori through the gastric mucus layer [1], and run-and-tumble dynamics of *Escherichia coli* in polymeric solutions [2]. Experimental studies have revealed important features of the movement of microorganisms in biological fluids [3]. In addition to experiments, mathematical modeling, analysis, and computational simulations are playing important roles in giving new insights on the mechanics behind small scale swimming in complex fluids [4,5].

Many biological fluids such as mucus and cytoplasm are mixtures of multiple components, which exhibit complicated non-Newtonian characteristics. The composition and rheology of the these mixtures have significant impact on the speed and efficiency of biological locomotion. On the other hand, the swimming motion is also greatly affected by physical boundaries and material interfaces near the swimmer. For example, *E. Coli* tends to exhibit circular trajectories close to a solid boundary [6]. Sperm cells near a liquid–solid interface swim slower than those near a liquid-gas interface [7]. Furthermore, some micro-swimmers may drastically enhance their motility by creating heterogeneous layers of fluids around them. For instance, self-propelling helical swimmers in shear-thinning fluids may move 50% faster than in a Newtonian fluid. The speedup is believed to come from the viscosity stratification around the swimmer [8]. Gastric mucin forms a layer of protective gel for the stomach lining against harmful bacteria. To swim through the mucus layer, *H. pylori* hydrolyzes ambient urea to produce ammonia. This causes an elevation of pH, and the transition of the local gastric mucus from a viscoelastic gel to a viscous fluid. Therefore, instead of boring its way across a mesh of polymer network, the bacterium essentially swims through a pocket of fluid surrounded by mucus gel [1]. Motivated by these experimental discoveries, there have been a number of theoretical analysis on the dynamics of swimmers near a plane surfactant-laden interface [9], in a viscous fluid enclosed by a Brinkman medium [10], within two layers of fluids separated by elastic deformable membranes [11] or by stationary interfaces [12]. By contrast, there are only few computational studies on locomotion in multi-component fluids, especially for problems involving dynamical fluid interfaces. In [13,14], different variations of the Immersed Boundary Method are proposed to simulate the interactions between elastic boundaries and a two-phase fluid mixture. However, no free interfaces are included in those studies.

In this paper, we investigate the classical Taylor’s swimming sheet problem near interfaces in a two-fluid environment, using the computational method developed in [13]. The mixture model provides a simple and unified framework for the study of swimming dynamics near interfaces in a multi-fluid mixture. Our simulation results reveal important characteristics of locomotion in heterogeneous fluid media, which are very different from those of swimming in a homogeneous environment without interfaces. In Section 2, the model equations and numerical methods are presented. The simulation results are presented in Section 3. The discussions about the results and potential extensions of the computational method are given in Section 4.

## 2. Model Equations and Numerical Method

### 2.1. Two-Fluid Mixture Model

In this work, the fluid medium surrounding a swimmer is described as a mixture of two immiscible fluids. We call the less viscous fluid in the mixture as the solvent phase (denote by s), and the more viscous fluid component as the network phase (denoted by n). Two-fluid models of this kind have been successfully used for the investigation of many biofluids such as blood clot, biofilm and cytoplasm [15,16]. At any spatial location x, the relative amounts of the two fluids are given by their volume fraction, $\theta_{\text{s}}(x,\text{t})$ and $\theta_{\text{n}}(x,t)$ for the solvent and network, respectively. The solvent and network fluids move with their own velocity fields, $u_{\text{s}}(x,\text{t})$ and $u_{\text{n}}(x,\text{t})$:

(1) $$\frac{\partial \theta_{\text{s}}}{\partial \text{t}}+\nabla \cdot \left(\theta_{\text{s}}u_{\text{s}}\right)=0,$$

(2) $$\frac{\partial \theta_{\text{n}}}{\partial \text{t}}+\nabla \cdot \left(\theta_{\text{n}}u_{\text{n}}\right)=0.$$

Since $\theta_{\text{n}}+\theta_{\text{s}}=1$, the sum of (1) and (2) gives the volume averaged incompressibility condition:

(3) $$\nabla \cdot \left(\theta_{\text{s}}u_{\text{s}}+\theta_{\text{n}}u_{\text{n}}\right)=0.$$

For a small Reynolds number, the force balance equations for the two fluids are given by:

(4) $$\nabla \cdot \left(\theta_{\text{s}}\sigma_{\text{s}}\right)-\theta_{\text{s}}\nabla \text{p}+\xi \theta_{\text{n}}\theta_{\text{s}}\left(u_{\text{n}}-u_{\text{s}}\right)+f_{\text{s}}=0.$$

(5) $$\nabla \cdot \left(\theta_{\text{n}}\sigma_{\text{n}}\right)-\theta_{\text{n}}\nabla \text{p}+\xi \theta_{\text{n}}\theta_{\text{s}}\left(u_{\text{s}}-u_{\text{n}}\right)+f_{\text{n}}=0,$$

Here, $\sigma_{\text{s}}$ and $\sigma_{\text{n}}$ are the viscous stress tensors for the solvent and network, respectively. $\xi \theta_{\text{n}}\theta_{\text{s}}\left(u_{\text{n}}-u_{\text{s}}\right)$ represents the frictional drag force between the two fluids due to relative motions, with ξ being the frictional coefficient. $f_{\text{s}}$ and $f_{\text{n}}$ are the force densities generated by immersed elastic structures (swimmer) on the two fluids. Their formulations will be discussed in the next section. $\sigma_{\text{s}}$ and $\sigma_{\text{n}}$ are taken to be those of Newtonian fluids:

(6) $$\sigma_{\text{s}}=\mu_{\text{s}}\left(\nabla u_{\text{s}}+\nabla {u_{\text{s}}}^{\text{T}}\right)+\left(\lambda_{\text{s}}\nabla \cdot u_{\text{s}}\right)\text{I},$$

(7) $$\sigma_{\text{n}}=\mu_{\text{n}}\left(\nabla u_{\text{n}}+\nabla {u_{\text{n}}}^{\text{T}}\right)+\left(\lambda_{\text{n}}\nabla \cdot u_{\text{n}}\right)\text{I}.$$

Here I is the identity tensor, $\mu_{\text{s}}$ and $\mu_{\text{n}}$ are the shear viscosities and $\lambda_{\text{s},\text{n}}+2\mu_{\text{s},\text{n}}/d$ are the bulk viscosities of the solvent and network (d is the space dimension). We choose $\lambda_{\text{s},\text{n}}=-\mu_{\text{s},\text{n}}$ so that the bulk viscosities in both fluids are zero. We nondimensionalize all model equations using the characteristic length scale of L=1μm, time scale of T = 1 second, and stress scale of $\mu_{\text{s}}/\text{T}$. The viscosity of the solvent $\mu_{\text{s}}$ is taken as the viscosity of water.

### 2.2. Immersed Boundary Method for Multi-Fluid Mixture

Immersed Boundary (IB) Method is a powerful computational approach for handling the dynamic interactions between fluid and immersed elastic structures [17]. The simplicity and robustness of the IB method lead to its numerous applications in biological problems [18–22]. In [13], a two-phase IB scheme is developed for the simulation of interactions between elastic structures and a two-fluid mixture. Here we briefly describe the basic elements of the method.

**Figure 1.**
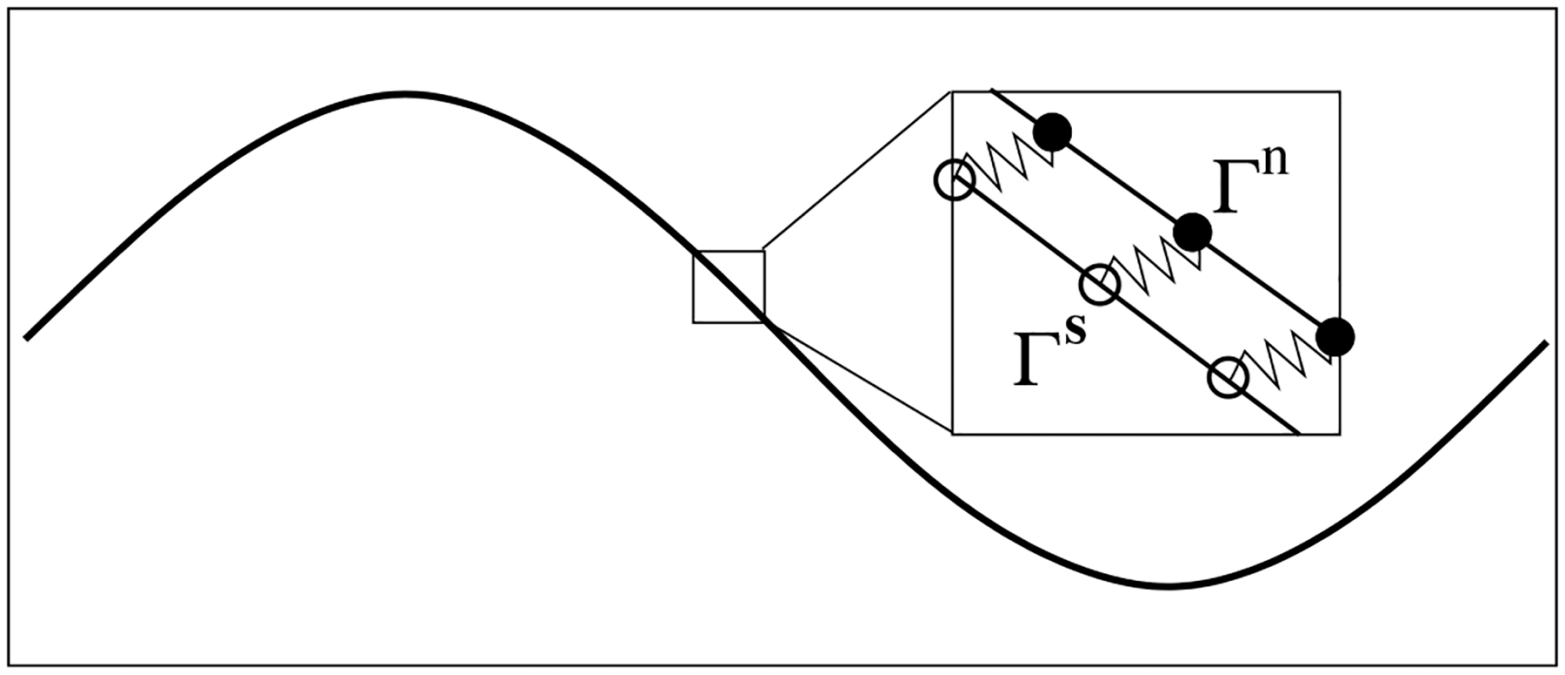
Dual IB representation of a swimmer. $•-X_{\text{n}}(\text{q},\text{t}),\circ -{\text{X}}_{\text{s}}(\text{q},\text{t})$.

In the two-phase IB scheme, an Eulerian description is used for the fluid variables such as the velocity and pressure. A Lagrangian description is used for the immersed structures. Each immersed structure is represented by two immersed boundaries, denoted by $\Gamma^{\text{n}}$ (network IB) and $\Gamma^{\text{s}}$ (solvent IB), respectively. As shown in Figure 1, the spatial locations of IB points on $\Gamma^{\text{n}}$ and $\Gamma^{\text{s}}$ are represented by the vector functions $X_{\text{n}}(\text{q},\text{t})$ and $X_{\text{s}}(\text{q},\text{t})$, respectively, with $X_{\text{n}}(\text{q},0)=X_{\text{s}}(\text{q},0)$. Here q is the parametrization variable and $X_{\text{n}}(\text{q},0)=X_{\text{s}}(\text{q},0)$. In this work, both the solvent and the network satisfy non-slip condition on the immersed structure. That is, the IB points on $\Gamma^{\text{n}}$ move with the local network velocity $u_{\text{n}}$. The IB points on $\Gamma^{\text{s}}$ move with the local solvent velocity $u_{\text{s}}$. To prevent $\Gamma^{\text{n}}$ and $\Gamma^{\text{s}}$ from separating, the pair of corresponding IB points on them is connected by a stiff spring with zero rest length. Penalty forces equal in magnitude with opposite directions are generated at these two points when their spatial locations are different. The penalty force density at $X_{\text{n}}$ is computed by $F_{\text{n}}^{\text{p}}=k_{p}\left(X_{\text{s}}-X_{\text{n}}\right)$, where $k_{p}$ is the penalty spring constant. The penalty force density $F_{\text{s}}^{\text{p}}$ at $X_{\text{s}}$ is computed in a similar way. In addition to the penalty forces, other types of forces are exerted at each IB point to simulate locomotion problems in this paper. First, to specify the internal elastic property of the swimmer, each IB point is connected by elastic springs to its two neighboring points on the same immersed boundary. Second, each IB point is connected by a stiff spring to a corresponding “tether” point with imposed track of motion. The movements of the tether point describe the specific gait of swimming.

In addition to the penalty force densities $F_{\text{n}}^{\text{p}}$ and $F_{\text{s}}^{\text{p}}$, we denote the densities of other force components (sum of elastic force and tether force) on the two boundaries by $F_{\text{n}}^{\text{o}}$ and $F_{\text{s}}^{\text{o}}$. Following the classical IB method, the coupling between the fluids and the immersed boundaries is through the integral relations:

(8) $$f_{\text{j}}^{\text{i}}(x,\text{t})=\int_{\Gamma^{\text{j}}}F_{\text{j}}^{\text{i}}(\text{q},\text{t})\delta \left(x-X_{\text{j}}(\text{q},\text{t})\right)\text{dq}$$

(9) $$\frac{\partial X_{\text{j}}(\text{q},\text{t})}{\partial \text{t}}=\int_{\Omega }u_{\text{j}}(x,\text{t})\delta \left(x-X_{\text{j}}(\text{q},\text{t})\right)\text{d}x$$

Here I = o, p and j = n, s. Ω is the fluid domain. $f_{\text{j}}^{\text{i}}(x,\text{t})$ represents the Eulerian force density at x contributed by the Lagrangian force $F_{\text{j}}^{\text{i}}.\delta (x)=\delta (\text{x})\delta (\text{y})$ is the two-dimensional Dirac delta function. Equation (8) describes how the Lagrangian forces at each IB point are transmitted to the corresponding fluid phase. Equation (9) reflects the fact that each IB object moves with the local fluid velocity. With Eulerian force densities defined above, the force densities in Equations (4) and (5) are calculated as:

(10) $$f_{\text{n}}=\theta_{\text{n}}f_{\text{n}}^{\text{o}}+\theta_{\text{n}}\theta_{\text{s}}f_{\text{n}}^{\text{p}},$$

and

(11) $$f_{\text{s}}=\theta_{\text{s}}f_{\text{s}}^{\text{o}}+\theta_{\text{n}}\theta_{\text{s}}f_{\text{s}}^{\text{p}}.$$

Within each fluid, the Eulerian contributions from $F_{\text{j}}^{\text{o}}$ is scaled by the volume fraction of that fluid. On the other hand, the penalty forces are scaled by the product of the volume fractions $\theta_{\text{s}}\theta_{\text{s}}$ after they are transmitted to each of the fluid. Therefore, no penalty force is generated if either of the volume fractions goes to zero. This also ensures that at each location, the total net penalty forces applied to the two fluids approximately add up to zero, provided that $\Gamma^{\text{n}}$ and $\Gamma^{\text{s}}$ are close enough.

### 2.3. Numerical Solutions

All fluid variables are discretized using a Cartesian grid, with constant grid space h. A MAC-type staggered computational grid is used for spatial discretization. Scalars are located at the grid centers and vectors are located at the grid edges. All spatial derivatives are discretized by centered finite difference. In addition to the discretized version of (1)–(5), the location of the IB points is updated by:

(12) $$X_{\text{n}}^{\text{k}+1}=X_{\text{n}}^{\text{k}}+\Delta \text{t}S_{\text{h}}^{*}\left(u_{\text{n}}^{\text{k}}\right),$$

(13) $$X_{\text{s}}^{\text{k}+1}=X_{\text{s}}^{\text{k}}+\Delta \text{t}S_{\text{h}}^{*}\left(u_{\text{s}}^{\text{k}}\right).$$

Here the symbol with superscript k represents the value of the corresponding variable at time step ${\text{t}}^{\text{k}}.S_{\text{h}}^{*}$ is the discretized version of the interpolation operator as defined in (9). The time iteration for the proposed numerical method can be summarized as following:

1. Based on the geometric configurations of IB curves $\Gamma^{\text{n}}$ and $\Gamma^{\text{s}}$ at ${\text{t}}^{\text{k}}$, compute the total elastic force densities $F_{\text{n}}$ and $F_{\text{s}}$ on them. Compute the Eulerian forces $f_{\text{n}}^{\text{k}}$ and $f_{\text{s}}^{\text{k}}$ by spreading $F_{\text{n}}$ to the network and $F_{\text{s}}$ to the solvent.
2. Solve discretized versions of (3)–(5) to get fluid velocities $u_{\text{n}},u_{\text{s}}$ at ${\text{t}}^{\text{k}}$.
3. Update the positions of the IB points at ${\text{t}}^{\text{k}+1}$ according to (12) and (13).
4. Compute $\theta_{\text{n}}$ at ${\text{t}}^{\text{k}+1}$ from discretized version of (2).
5. Repeat step 1 at next time level ${\text{t}}^{\text{k}+1}$.

Details about the computational algorithm are given in [13].

## 3. Results

### 3.1. Problem Setup

The problem we investigate is motivated by the classical Taylor’s swimming sheet problem [23]. The swimmer is modeled as an infinite extensible thin sheet, which is a curve in the two dimensional plane. In the reference frame moving with its swimming speed, the sheet has a waving profile:

(14) $$\text{y}=\text{b}\;\text{sin}\left(\text{kx}-\omega \text{t}\right).$$

In all 2D plots, the x-axis and y-axis are along the horizontal and vertical directions, respectively. We choose k=ω=2π. The computational domain is over the rectangular region [0, 1] × [−1.5, 1.5]. Periodic boundary conditions are imposed in the x-direction for all model variables to simulate the movement of an infinite swimmer. No-slip conditions are imposed for all velocity components at y = ±1.5. The size of the computational grid is 128 × 384. A constant time step $\Delta \text{t}=10^{-4}$ is used for all simulations. The swimming sheet is represented by two IB objects ($\Gamma^{\text{n}}$ and $\Gamma^{\text{s}}$) each with 256 IB points. The swimming speed of the sheet is computed by averaging the x-velocity of all the IB points over one wave period. We assume a steady value is obtained when the swimming speed varies less than 5% between two consecutive periods.

Since the main focus of this paper is to study locomotion within layers of fluids separated by interfaces, we choose the initial profile of the network volume fraction $\theta_{\text{n}}$ as a piece-wise constant function in the y-direction:

$$\theta_{\text{n}}(\text{x},\text{y},\text{t}=0)=\left\{\begin{array}{ll} \theta_{\text{n}}^{\text{in}} & \text{if}\left|\text{y}\right|\leq \text{H},\\ \theta_{\text{n}}^{\text{out}} & \text{if}\left|\text{y}\right|>\text{H}. \end{array}\right.$$

Here the value of H defines the initial location of the fluid interface. $\theta_{\text{n}}^{\text{i}n}$ is the network volume fraction in the thin fluid layer in direct contact with the swimmer. This inner layer is surrounded by a bulk fluid with a network volume fraction of $\theta_{\text{n}}^{\text{out}}$. Notice that the location of the fluid interfaces may change due to the movement of the swimming sheet. To solve numerically the equations of motion with free boundaries between different phases of materials, interface tracking methods can be used. The location of the interface is followed explicitly, and different PDEs coupled by matching conditions are solved on the each side of the interface [24]. In this work, we use an interface capturing strategy [25] in which the same two-fluid Equations (3) and (4) are solved everywhere throughout the domain. The fluid interface is implicitly represented by the spatial distribution of the volume fraction $\theta_{\text{n}}$, which may be regularized numerically to make computational solutions feasible. There is no need to explicitly track the dynamic interface and enforce the interface conditions. Because of the capturing strategy and numerical smearing, our simulations essentially approximate the fluid interface by a spatial region (in 2D) over which the values of $\theta_{\text{n}}$ exhibit sharp transitions.

### 3.2. Comparison with Analytic Solutions

In this section, we illustrate the accuracy of the numerical algorithm by comparing our simulation results with analytical solutions. In [11,12], the swimming speed is calculated for an infinite waving sheet in a viscous two-fluid domain, where a low-viscosity fluid layer near the swimmer is surrounded by a more viscous bulk fluid. For reasonable comparisons with asymptotic solutions, we choose b = 0.012 so that bk ≪ 1. As the initial profile, we set $\theta_{\text{n}}^{\text{i}n}=0$ and $\theta_{\text{n}}^{\text{o}ut}=1$ so that the inner fluid layer only contains solvent and the bulk fluid only contains network. each fluid layer only contains a single fluid. Note that the force balance Equations (4) and (5) become degenerate over the region with $\theta_{\text{s}}=0$ or $\theta_{\text{n}}=0$. To avoid this difficulty, we regularize the discrete equations by temporarily adding a small positive constant $\delta \theta_{\text{n}}=1.0e-5$ to $\theta_{\text{n}}$ throughout the entire domain and solve the same set of two-fluid equations everywhere. The value of $\theta_{\text{s}}$ is reduced by the same constant to maintain the condition $\theta_{\text{n}}+\theta_{\text{s}}=1$. A large frictional coefficient $\xi =10^{10}$ is chosen so that the two velocity fields $u_{\text{n}}$ and $u_{\text{s}}$ are approximately the same.

#### 3.2.1. Swimming Near Non-Deformable Fluid Interface

**Figure 2.**
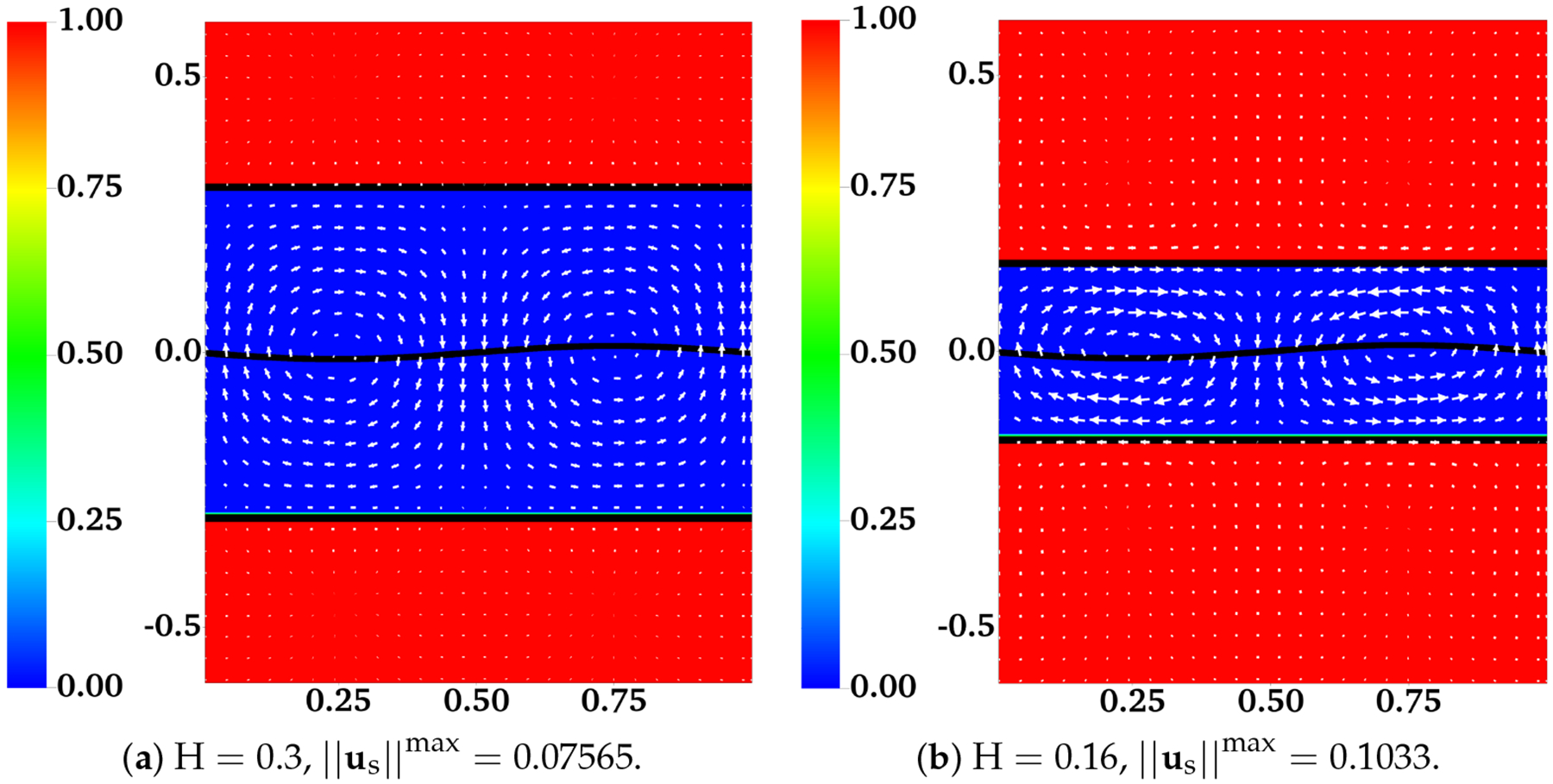
Distribution of network volume fraction $\theta_{\text{n}}$ and fluid velocity $u_{\text{s}}$ at t = 0.5 for different H. All vectors have the same scale. The viscosity ratio between the two fluids is β=0.5.

**Figure 3.**
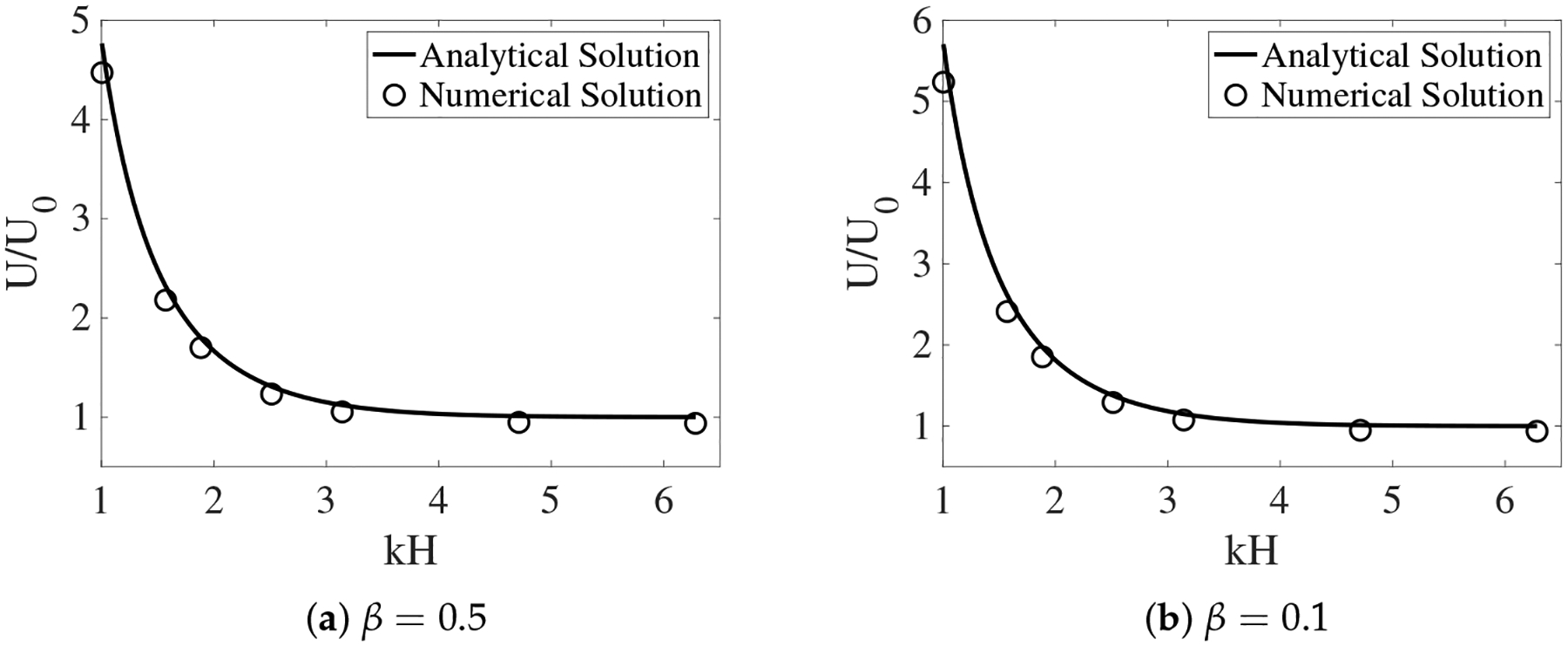
The scaled swimming speed vs. the nondimensional interface height kH for different β. The fluid interfaces are non-deformable. The analytical solutions are from [12].

In [12], the interface between the two fluids remains flat. Across the interface, the y-component of the fluid velocities is zero. The x-component of the fluid velocities is continuous. To enforce such interface conditions, we introduce two horizontal IB walls initially located at y = ±H. Each wall is composed of two IB objects as described in the previous section. To approximate a flat interface during the simulation, every IB point (x, y) on the walls is connected by a stiff spring to a tether point at (x, H). Penalty forces are generated at IB points on the walls with y ≠ H caused by vertical motions of the fluids. The simulation setup is shown in Figure 2. In the plot, the distributions of $\theta_{n}$ and $u_{\text{s}}$ (as well as $u_{\text{n}}$) are shown for two simulations with different interface locations at t = 0.5. The viscosity ratio is set to $\beta =\frac{\mu_{\text{s}}}{\mu_{\text{n}}}=0.5$. The horizontal black lines in the plot are the IB walls that simulate fluid interfaces. The black curves in the middle represent the swimming sheet. For the simulation in which the fluid interfaces are far away from the sheet (Figure 2a, H = 0.3), the largest fluid velocity (along the y-direction) appears near the swimmer. The value of ${\left\|u_{\text{s}}\right\|}^{\text{max}}$ is close to the largest vertical velocity of the sheet. As shown in Figure 2b, the largest fluid velocity appears away from the swimmer along the x-direction in the simulation with H = 0.16. Due to the confinement effect from the flat interfaces, the counter-rotating vortices are much stronger for the simulation with smaller H. The net effect is that the waving sheet closer to the interfaces tends to move faster along the negative x-axis. In Figure 3a, b, the simulated swimming speed (scaled by the swimming speed in a single phase Newtonian fluid U_0_) is plotted as a function of the nondimensional interface height kH for two sets of simulations with viscosity ratios β=0.5 and β=0.1, respectively. For a fixed value of kH, the sheet swims faster with more significant viscosity differences between the two fluid layers. The simulations agree well with the 2nd order analytic results in [12].

#### 3.2.2. Swimming Near Deformable Fluid Interface

**Figure 4.**
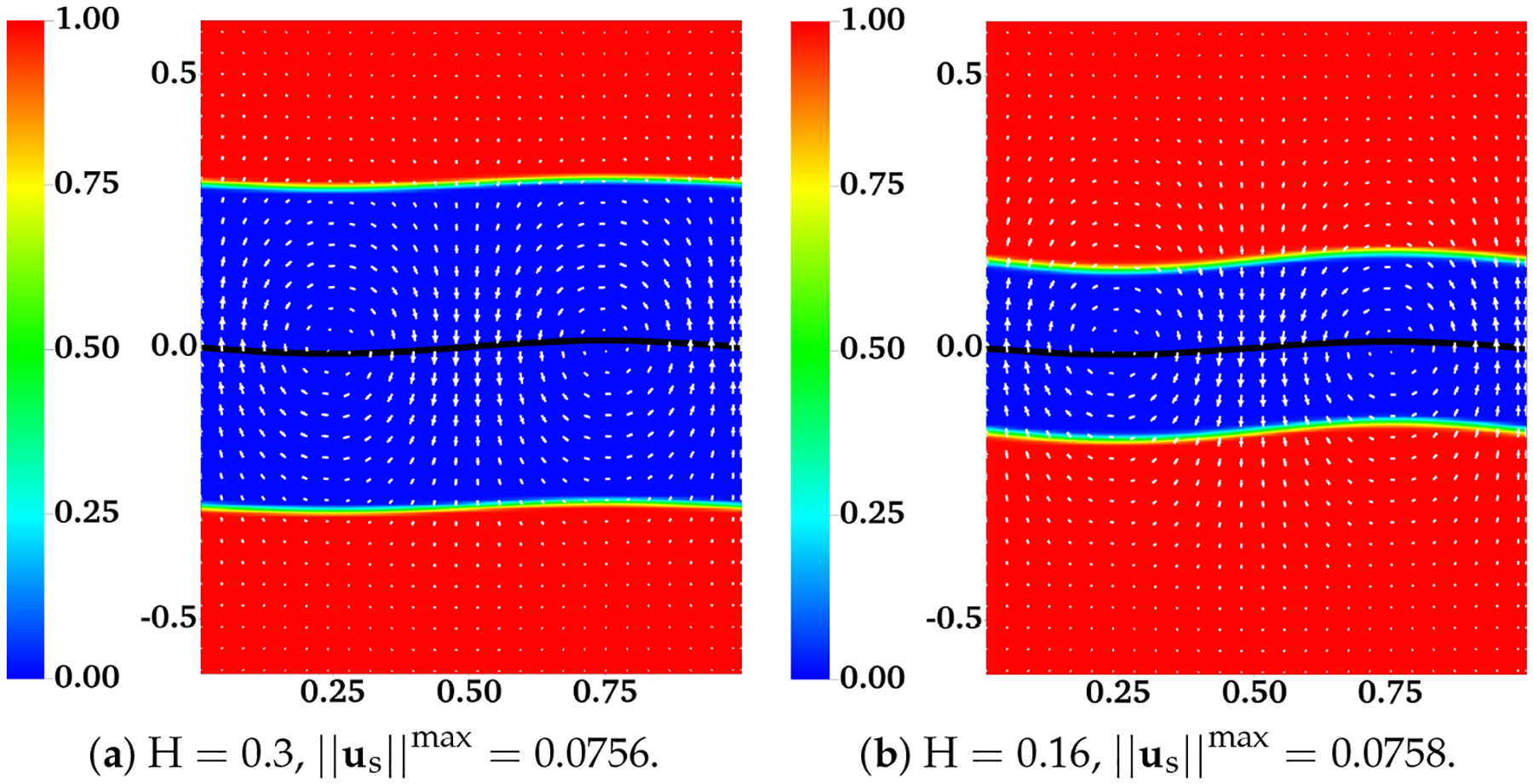
Distribution of network volume fraction $\theta_{\text{n}}$ and fluid velocity $u_{\text{s}}$ at t = 0.5 for different H. All vectors have the same scale. The viscosity ratio between the two fluids is β=0.5.

**Figure 5.**
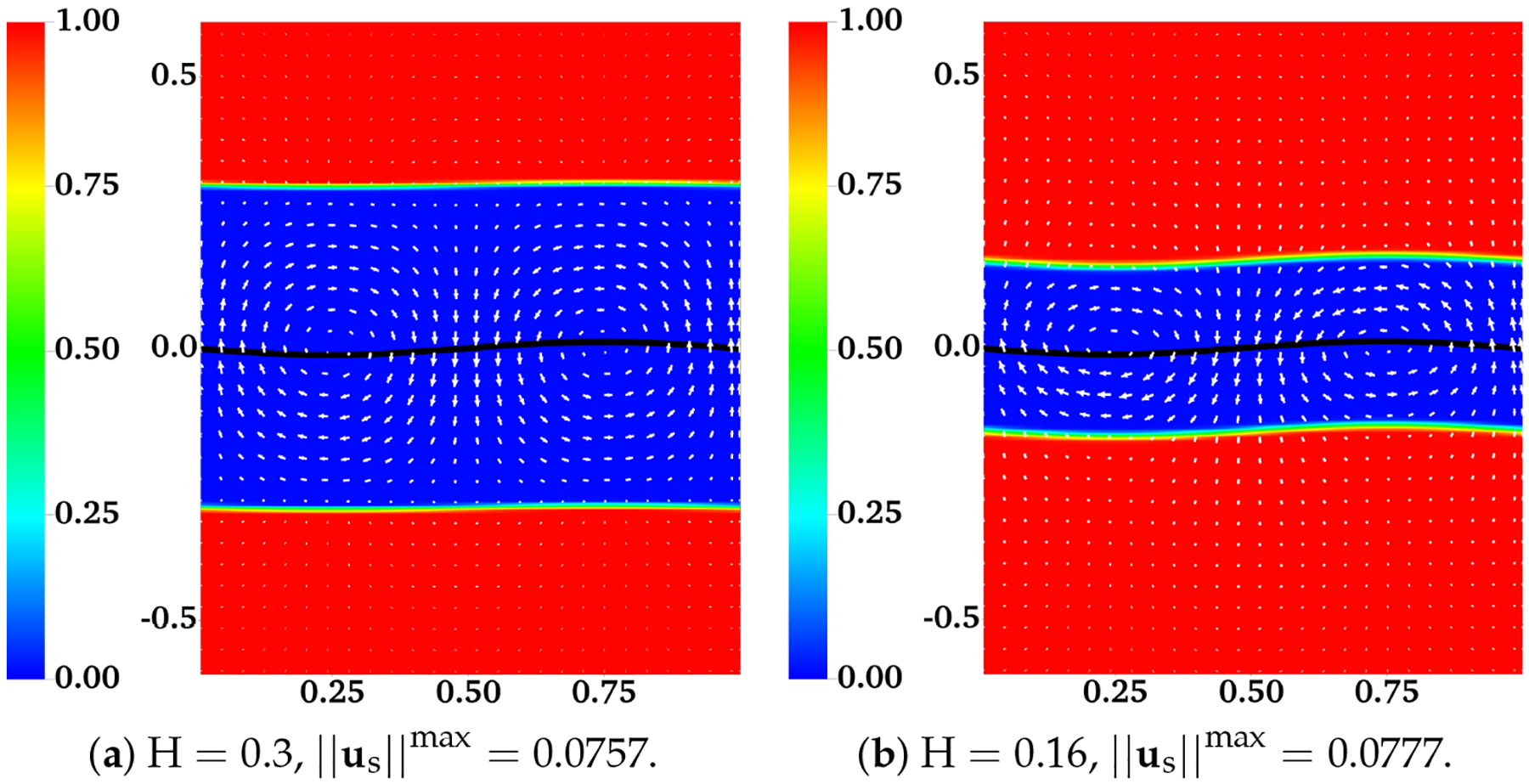
Distribution of network volume fraction $\theta_{\text{n}}$ and fluid velocity $u_{\text{s}}$ at t = 0.5 for different H. All vectors have the same scale. The viscosity ratio between the two fluids is β=0.1.

**Figure 6.**
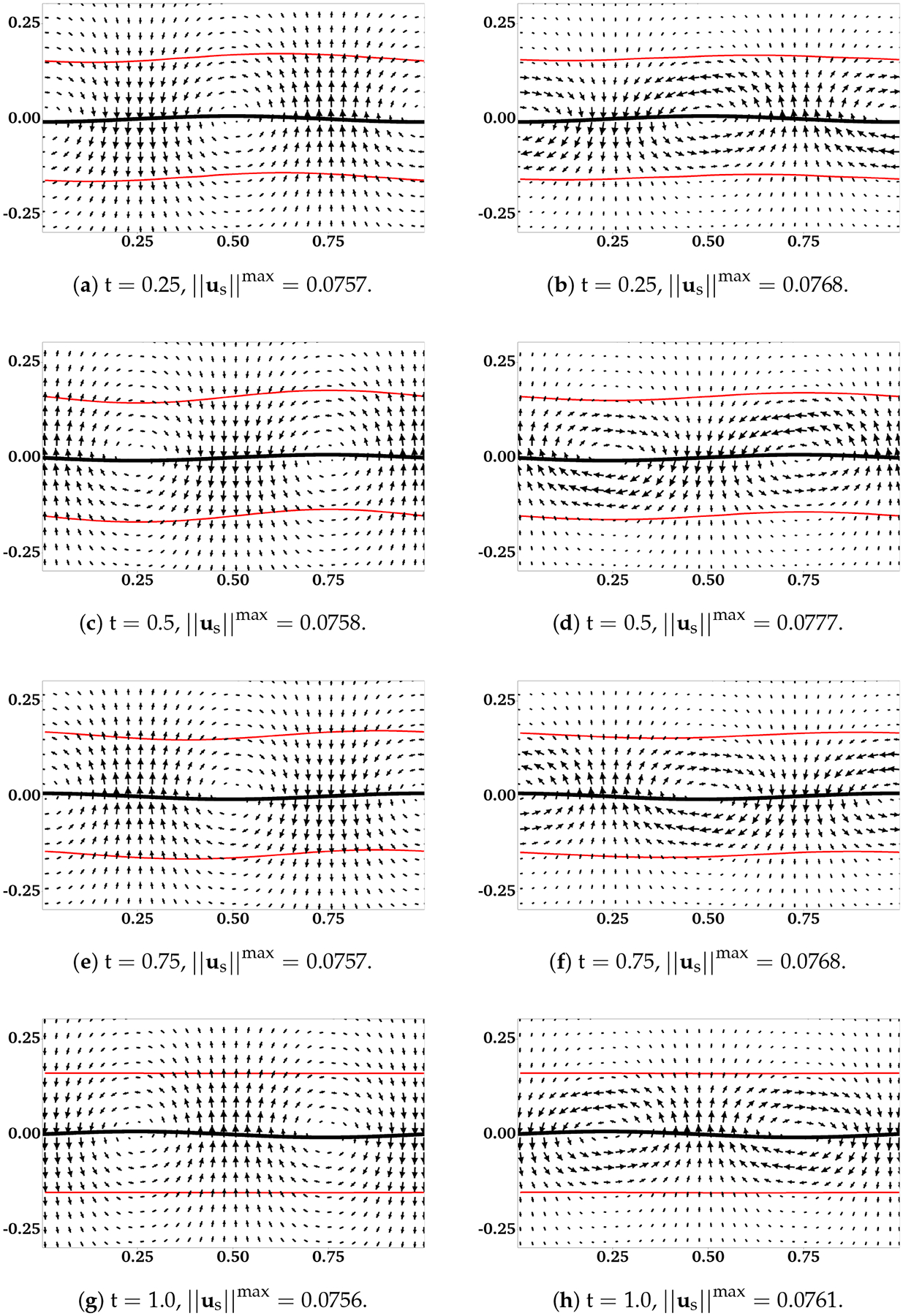
H = 0.16. Distributions of $u_{\text{s}}$, the profiles of swimming sheet (black curves) and fluid interfaces (red curves) for different β. (**a**,**c**,**e**,**g**) are for β=0.5. (**b**,**d**,**f**,**h**) are for β=0.1. All vectors have the same scale.

**Figure 7.**
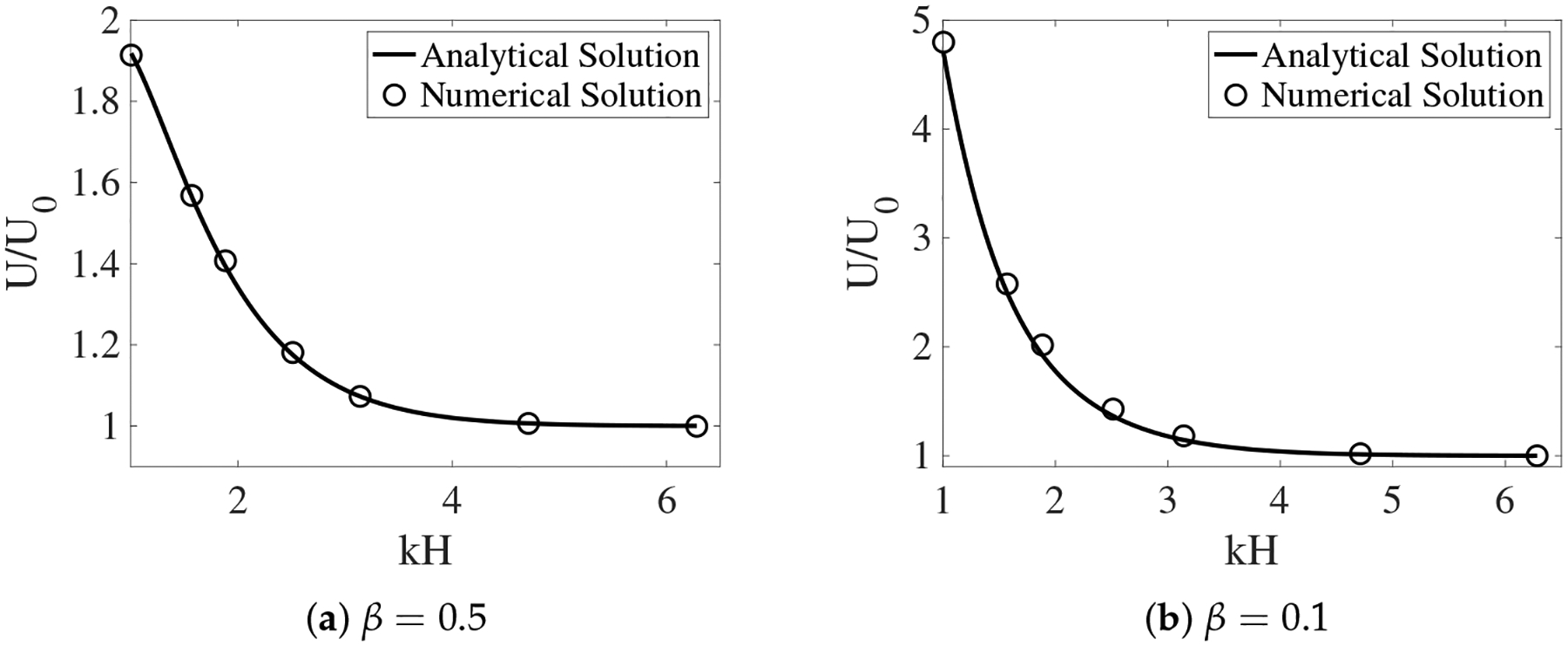
The scaled swimming speed vs. the nondimensional interface height kH for different β. The fluid interfaces are deformable. The analytical solutions are from [11].

The assumption that the fluid interfaces remain flat near a waving sheet is only valid for large H or small β. In [11], the locomotion of an infinite swimmer close to a deformable interface between two viscous fluids is investigated analytically. The interface condition is given by the continuity of stress and velocity. To illustrate the accuracy of our interface-capturing scheme, we remove the IB walls at y=±H and redo the simulations as described in the previous section. In Figures 4 and 5, the distributions of $\theta_{\text{n}}$ and $u_{\text{s}}$ are plotted for simulations with β=0.5 and β=0.1, respectively. As an indication of the interface locations, the jump in $\theta_{\text{n}}$ is slightly smeared over 1 – 2 mesh blocks at t = 0.5 relative to its initial sharp profile. The interfaces deform from their horizontal configurations due to the waving motion of the swimmer. For interfaces closer to the swimmer, the deformations are more significant. Comparing Figure 5 with Figure 4, there are less interface deformations in the simulation with a smaller viscosity ratio β. Because of the smearing effect, we choose to visualize the fluid interfaces using the contour lines of $\theta_{\text{n}}=0.5$, a value in the middle of the network volume fractions within the inner and outer layers of fluids. The swimming sheet, the “fluid interfaces”, and the fluid velocity are plotted in Figure 6 for two simulations with β=0.5 and β=0.1 at different time. The initial swimmer-interface distance is H = 0.16. It can be noticed that for the simulation with a smaller viscosity ratio (right column of Figure 6), the less deformed fluid interfaces provide more effective confinements to the swimmer. Thus, the fluid velocity exhibits stronger counter-rotating vortices than those in the simulation with a larger β (left column of Figure 6). By the time at t = 1.0 (which is exactly one wave period), all fluid interfaces return approximately back to their initial horizontal positions. In Figure 7, the simulated swimming speed is compared with the 2nd order analytical result in [11]. The agreement is very good.

#### 3.2.3. Force Analysis

For a Stokes swimmer, the swimming speed is determined by a balance of thrust and drag forces [26]. Here the thrust ${\text{F}}_{T}$ is defined as the anchoring force applied so that the undulating sheet is prevented from swimming. The drag is the force $F_{D}$ required to tow the sheet with a frozen shape at the swimming speed U. Due to linearity of Stokes flow, the drag force can be expressed as ${\text{F}}_{D}(\text{U})=\gamma \text{U}$, where the magnitude of the drag coefficient γ is equal to ${\text{F}}_{D}(1)$. To compute the thrust force, we carry out simulations in which tether points are used to prevent IB points on a waving sheet from moving in the x direction. ${\text{F}}_{T}$ is computed as the total tethering force on the sheet, averaged over one wave period. Similarly, for simulations in which IB points on a frozen sheet are connected to tether points moving with velocity of 1 along the x direction, γ can be calculated from the time averaged towing force on the sheet. From the force balance equation ${\text{F}}_{T}+{\text{F}}_{D}=0$, the swimming speed is given by $\text{U}=-\frac{{\text{F}}_{T}}{\gamma }$. To confirm the accuracy of the thrust and drag calculation from the simulations, we list the simulated results in Tables 1 and 2 for non-deformable and deformable interfaces, respectively. The viscosity ratio is β=0.5 for all calculations. As seen from the tables, the ratio between the thrust force and the drag coefficient from the simulations is very close to the swimming speed of the sheet.

**Figure 8.**
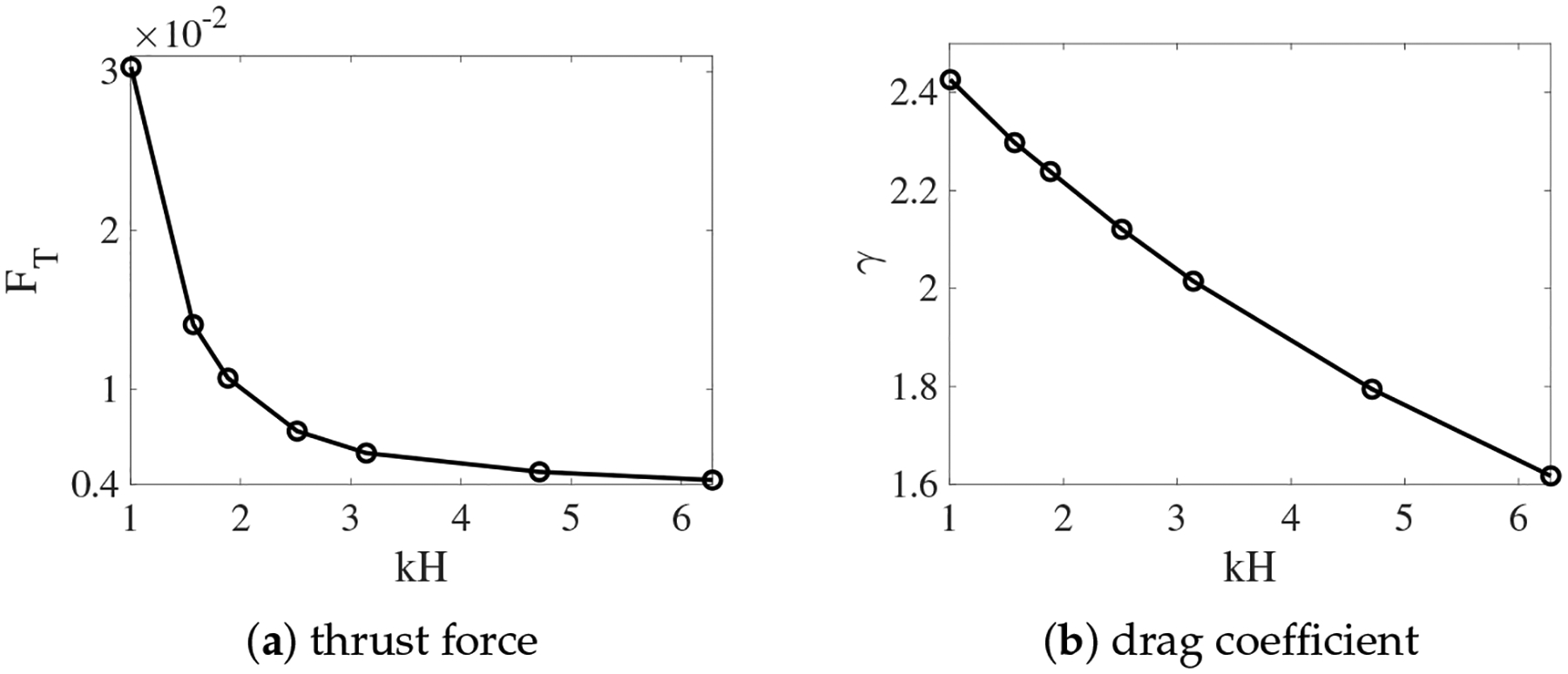
Thrust and drag coefficient vs. the nondimensional interface height kH. β=0.5. The fluid interfaces are not deformable.

**Figure 9.**
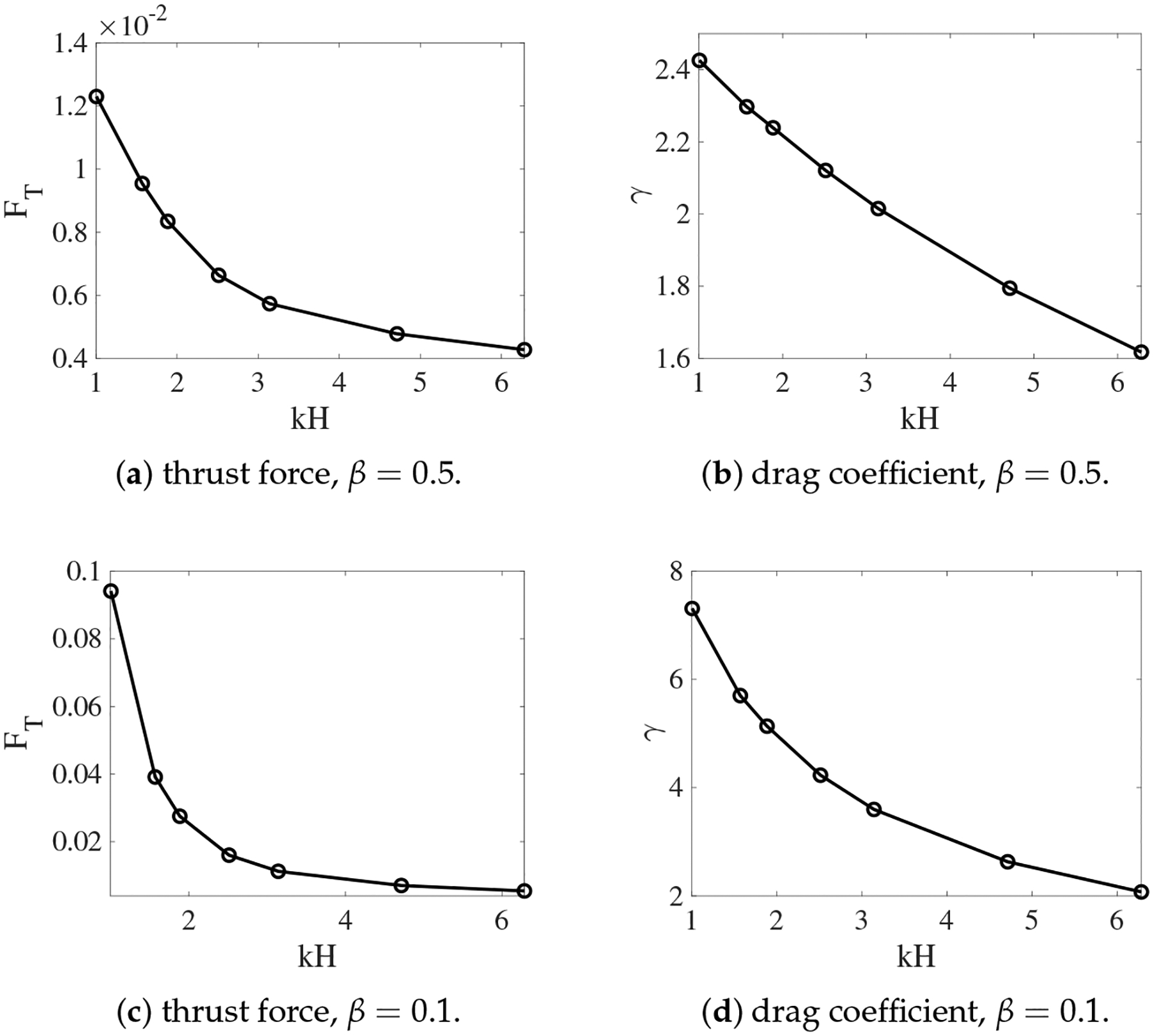
Thrust and drag coefficient vs. the nondimensional interface height kH for different β. The fluid interfaces are deformable.

In Figure 8, we plot the thrust force and the drag coefficient for different values of kH from the simulations with non-deformable interfaces. Both ${\text{F}}_{T}$ and γ increase as the fluid interfaces get closer to the swimmer. The variation is approximately linear for γ and superlinear for ${\text{F}}_{T}$. As the result, swimmers near non-deformable fluid interfaces always move faster than those in a single fluid, as observed in Figure 3. The enhancement of the swimming speed is more dramatic at smaller values of kH, where the thrust force increases more significantly. For simulations in which the interfaces are deformable, the values of ${\text{F}}_{T}$ and γ are plotted in Figure 9 for β=0.5 and 0.1. Comparing Figure 9a,b with Figure 8, we see that for a fixed viscosity ratio, the drag coefficient is almost identical for simulations with non-deformable and deformable interfaces. On the other hand, close to kH = 1, the thrust force is much smaller in the simulations with deforming interfaces. Physically, a swimmer can move much faster near non-deformable interfaces than near deformable ones, due to the larger thrust force from the stronger confinement effect. Comparing the thrust and drag curves for different values of β in Figure 9, it is noticed that for the same value of kH, both ${\text{F}}_{T}$ and γ increase with the decrease of β. Compared with the drag coefficient, the rate of increase is higher for the thrust force. As the result, the sheet can move faster within two fluid layers where there is a greater viscosity difference.

### 3.3. Swimming Near Deformable Interface in Fluid Mixtures

For all simulations presented so far, the continuum medium on each side of the interface is modeled as a single phase viscous fluid. Such models may not be suitable for some applications. For example, many biological fluids such as gastric mucus are mixtures composed of a polymer network immersed in a fluid solvent. The mixture can not be adequately described as a single phase homogeneous medium if the composition of the mixture has large spatial variations, or when there is significant relative motions between different components in the mixture. In this section, we investigate problems for which analytical solutions are not available. Specifically, we study the dynamics of the swimming sheet in mixtures of fluids separated by free interfaces. The main focus is on the effect of fluid composition and frictional drag on the swimming motion. A relatively large wave amplitude b = 0.048 is chosen for the swimming sheet. At the initial time, the network volume fraction in the bulk fluid away from the swimmer is set to $\theta_{\text{n}}^{\text{out}}=0.8$. The value of $\theta_{\text{n}}^{\text{in}}$ for the mixture around the swimmer is varied between simulations. We also vary the frictional coefficient ξ between the network and the solvent to investigate the influence of phase separation on locomotion. For all simulations in this section, the viscosity ratio between the solvent and the network is β=0.25.

**Figure 10.**
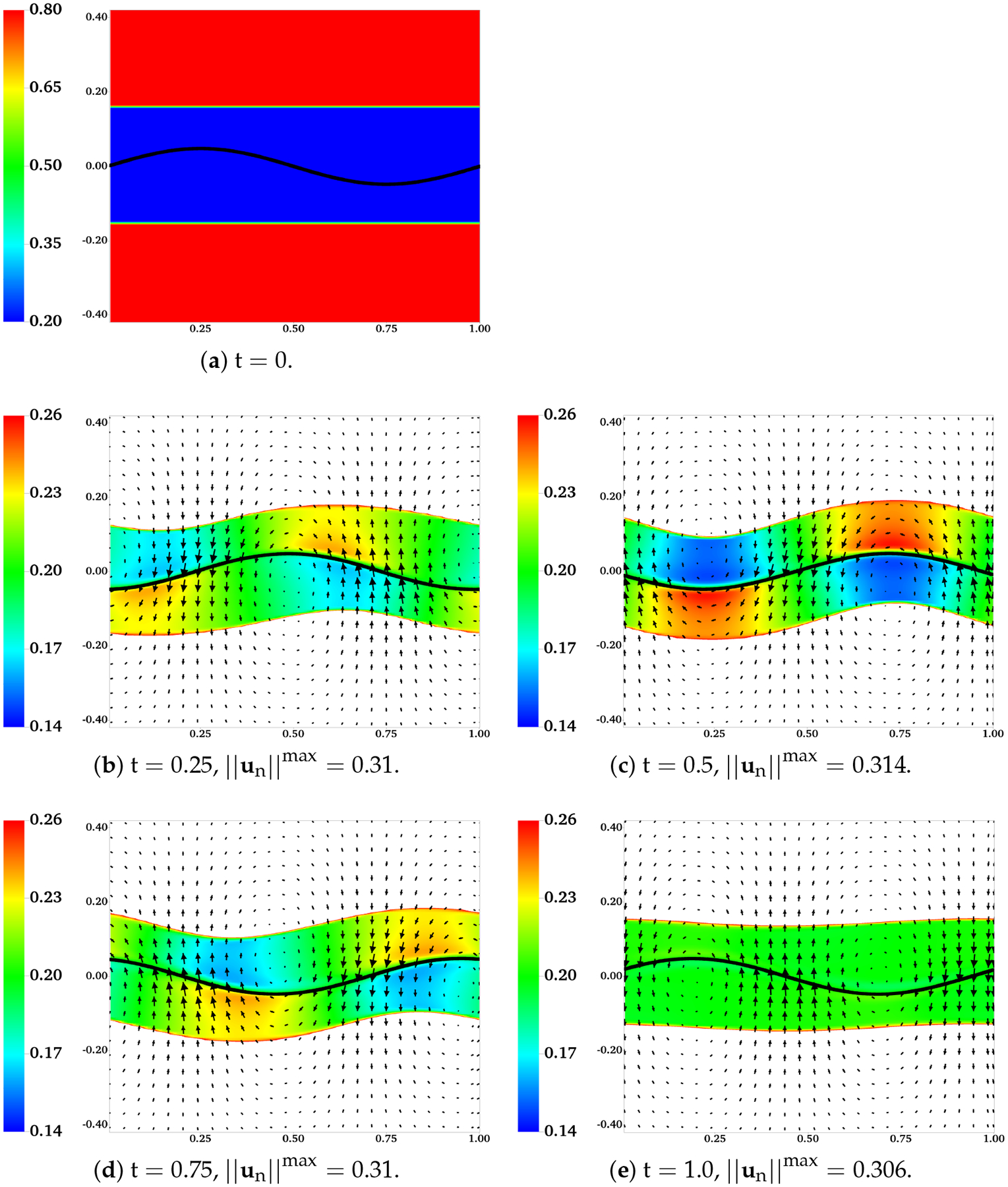
H = 0.16, $\beta =0.25,\xi =100,\theta_{\text{n}}^{\text{i}n}=0.2$, and $\theta_{\text{n}}^{\text{out}}=0.8$. Distributions of $\theta_{\text{n}}$ and $u_{\text{n}}$ at different time. All vectors have the same scale.

**Figure 11.**
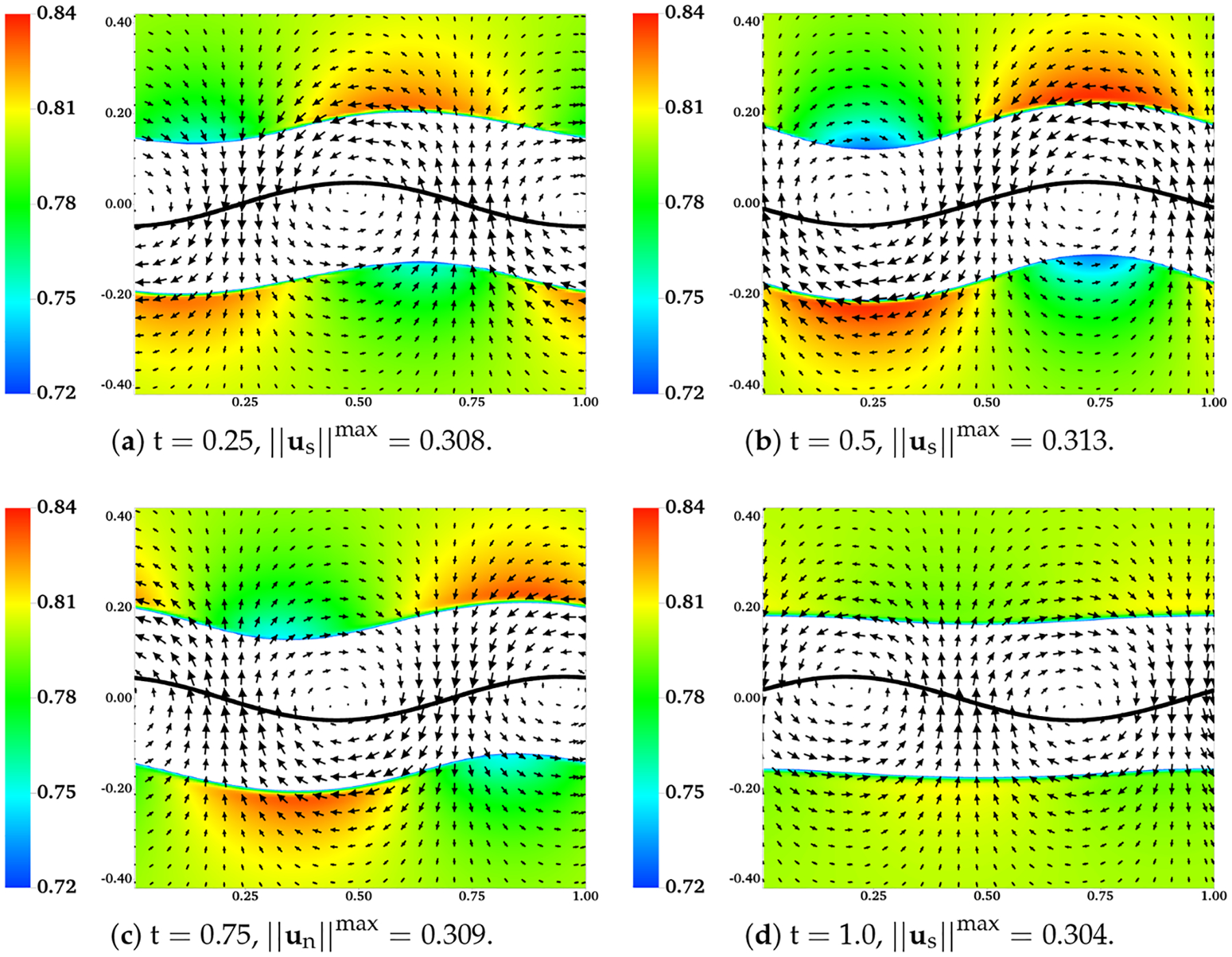
H=0.16, $\beta =0.25,\xi =100,\theta_{\text{n}}^{\text{i}n}=0.2$, and $\theta_{\text{n}}^{\text{out}}=0.8$. Distributions of $\theta_{\text{n}}$ and $u_{\text{s}}$ at different time. All vectors have the same scale.

In Figure 10, the distributions of $\theta_{\text{n}}$ and $u_{\text{n}}$ are plotted at different time for the simulation in which H = 0.16, ξ=100, and $\theta_{\text{n}}^{\text{i}n}$ is set to 0.2 at the initial time. To give a better contrast, white color is used to fill regions with $\theta_{\text{n}}>\theta_{\text{n}}^{\text{i}n,max}$, where $\theta_{\text{n}}^{\text{i}n,max}=0.26$ is the largest value of $\theta_{\text{n}}$ observed near the sheet during the simulation. In Figure 10b–e, the boundaries of the colored region (contour lines of $\theta_{\text{n}}=\theta_{\text{n}}^{\text{i}n,\text{max}}$) are referred to as the “fluid interfaces”. As time progresses, spatial inhomogeneities in $\theta_{\text{n}}$ and deformations of fluid interfaces appear with the waving motion of the sheet. Compared with the plots at t = 0.25 and t = 0.75, there are more significant inhomogeneities in the spatial distribution of $\theta_{\text{n}}$ at the half wave period t = 0.5. Interestingly, it is also at t = 0.5 that the fluid interfaces and the swimmer have approximately the same phase. At one wave period t = 1.0, the distribution of network volume fraction roughly returns to its initial value of 0.2 over the entire inner layer. At t = 1.0, although the fluid interfaces do not fully return to their horizontal positions, their vertical deformations are the smallest within the wave period. Relative to the results at earlier time, the network velocity $u_{\text{n}}$ tends to have smaller x-components at t = 1.0. The distributions of $\theta_{\text{n}}$ over the outer layers of fluid and the solvent velocity $u_{\text{s}}$ are plotted in Figure 11. Here white color is used to fill the region where $\theta_{\text{n}}>\theta_{\text{n}}^{\text{i}n,\text{out}}$, with $\theta_{\text{n}}^{\text{i}n,\text{out}}=0.72$ being approximately the smallest value of $\theta_{\text{n}}$ in the outer layer. The fluid interfaces and $\theta_{\text{n}}$ in the plot exhibit similar periodic patterns in time as observed in Figure 10. Notice that due to the numerical smearing, there is a small vertical displacement (about 2 grid blocks) between the fluid interfaces visualized in Figures 10 and 11. At one wave period t = 1.0, x-components of the solvent velocity have smaller magnitude than those at earlier time. Due to its low viscosity, the solvent has greater motion than the network in the vicinity of the swimmer.

**Figure 12.**
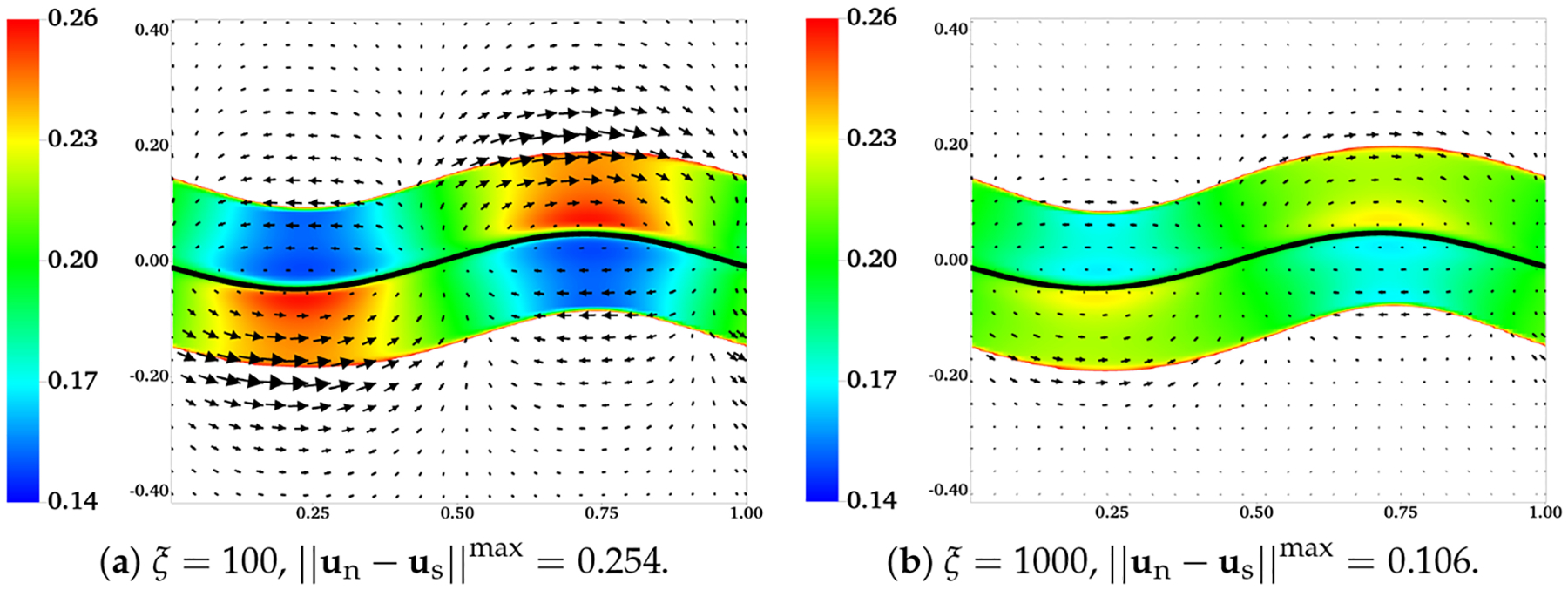
Effect of frictional coefficient: H=0.16, $\beta =0.25,\theta_{\text{n}}^{\text{i}n}=0.2$, and $\theta_{\text{n}}^{\text{out}}=0.8$. Distributions of $\theta_{\text{n}}$ and velocity difference $u_{\text{n}}-u_{\text{s}}$ at t = 1.0. All vectors have the same scale.

**Figure 13.**
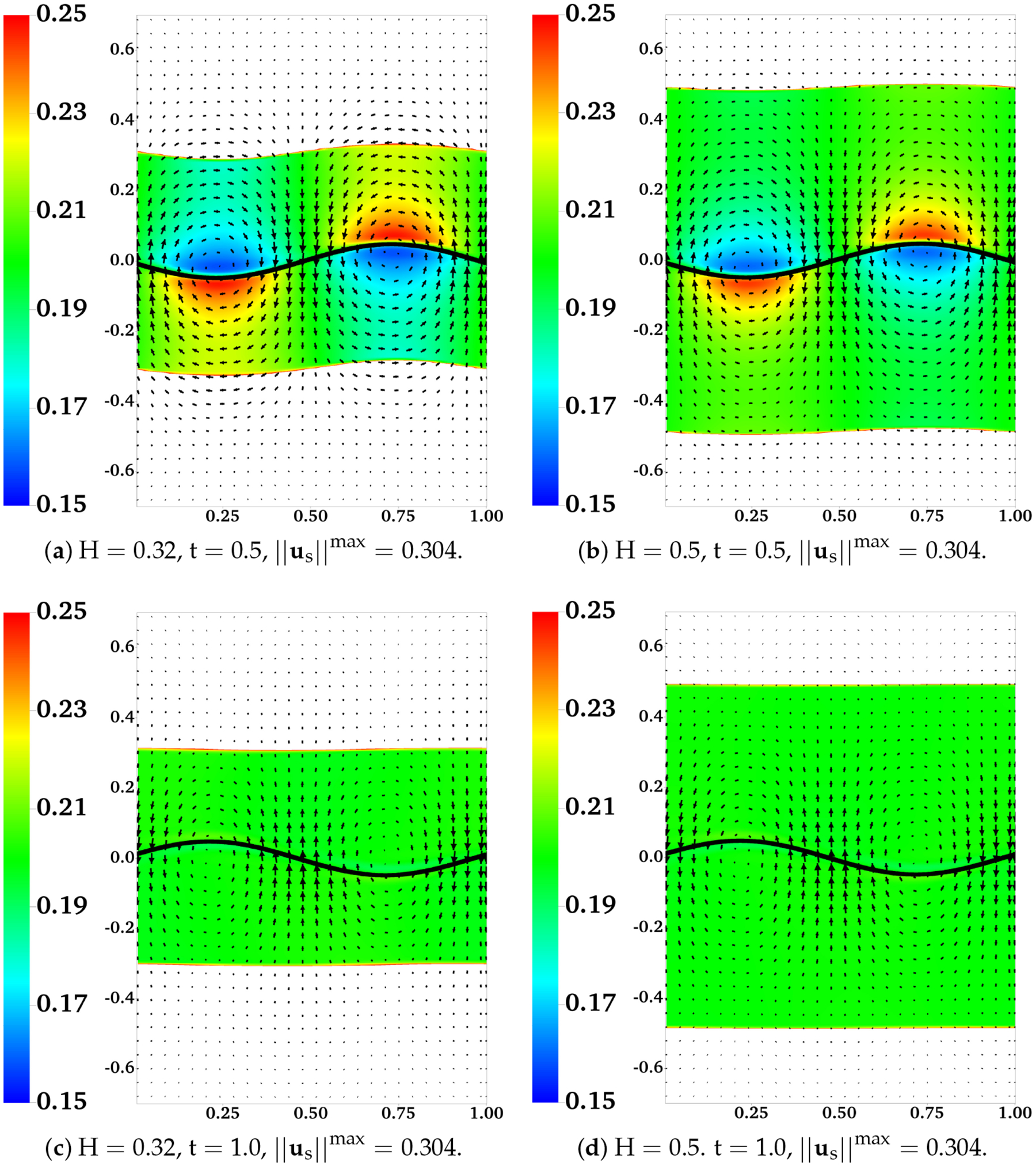
Effect of interface location H: $\beta =0.25,\xi =100,\theta_{\text{n}}^{\text{i}n}=0.2$, and $\theta_{\text{n}}^{\text{out}}=0.8$. Distributions of $\theta_{\text{n}}$ and $u_{\text{s}}$ at different time. All vectors have the same scale.

In Figure 12, we plot the distributions of $\theta_{\text{n}}$ and the velocity difference $u_{\text{n}}-u_{\text{s}}$ for two simulations with ξ=100 and ξ=1000, at t = 0.5. With a 10-fold increase of the frictional coefficient, there is a drastic reduction in both the extent of phase separation and the relative motion between the two fluids. The region with the largest velocity difference is near the fluid interfaces. A close look at the plot indicates that the interface deformation is larger in the simulation with a larger frictional coefficient (not shown). In Figure 13, we show the distributions of $\theta_{\text{n}}$ and $u_{\text{s}}$ from two simulations with H = 0.32 and H = 0.5 at t = 0.5 and t = 1. Regions with $\theta_{\text{n}}>0.25$ are filled with white color. Comparing the plots in Figure 13a,b, we see that with the locations of the fluid interfaces further away from the swimmer, both the interface deformation and the extent of phase separation are reduced. The vortices of $u_{\text{s}}$ for the simulation with H = 0.32 are stronger than than those in the simulation with H = 0.5. As indicated in Figure 13c,d, at one wave period t = 1, the distributions of $\theta_{\text{n}}$ return approximately to their initial profile for both simulations. Furthermore, at this moment, all fluid interfaces are horizontal approximately, especially for the simulation with the larger value of H.

**Figure 14.**
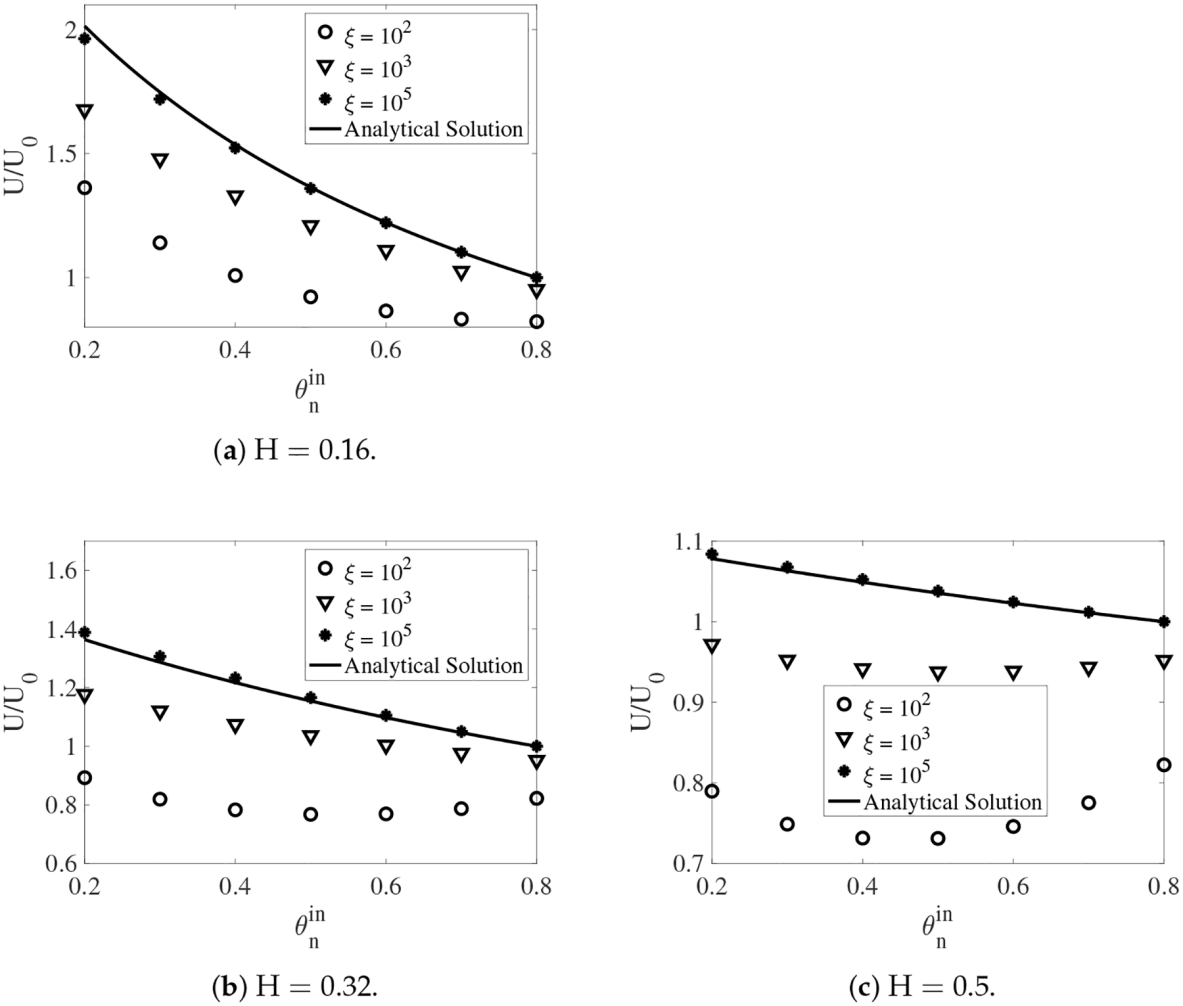
The scaled swimming speed vs. $\theta_{\text{n}}^{\text{i}n}$ for different H and ξ.β=0.25 and $\theta_{\text{n}}^{\text{out}}=0.8$. The analytical solutions are from [11].

In Figure 14, the scaled swimming speed $\frac{𝒰}{{𝒰}_{0}}$ is plotted as a function of the network volume fraction $\theta_{\text{n}}^{\text{i}n}$ for H = 0.5, H = 0.32, and H = 0.16. The value of $\theta_{\text{n}}^{\text{o}ut}$ is 0.8 for all simulations. For all values of H, the swimming speed always increases with the increase of the frictional coefficient for a given $\theta_{\text{n}}^{\text{i}n}$. This is consistent with the conclusion from [27], in which the swimming sheet problem is investigated in a two-fluid mixture without interfaces. In the simulations with the largest drag of $\xi =10^{5}$, the network and the solvent move approximately with the same velocity. Therefore, the inner fluid layer behaves like a single phase fluid with the effective viscosity of $\mu_{\text{e}ff}^{\text{i}n}=\theta_{\text{n}}^{\text{i}n}\mu_{\text{n}}+\left(1-\theta_{\text{n}}^{\text{i}n}\right)\mu_{\text{s}}$. Similarly, the effective viscosity of the outer layer is given by $\mu_{\text{e}ff}^{\text{o}ut}=\theta_{\text{n}}^{\text{o}ut}\mu_{\text{n}}+\left(1-\theta_{\text{n}}^{\text{o}ut}\right)\mu_{\text{s}}$. Based on the viscosity ratio $\beta =\frac{\mu_{\text{e}ff}^{\text{i}n}}{\mu_{\text{e}ff}^{\text{out}}}$, we can use the analytical formula from [11] to compute the swimming speed. The results are plotted as the solid black curves in Figure 14. Despite of the relatively large amplitude of the waving sheet and large interface deformations, the simulation results agree very well with the analytical solutions. Notice that for the simulations with the largest drag, the swimming speed in a single phase fluid is recovered $\left(\frac{𝒰}{{𝒰}_{0}}\approx 1\right)$ when $\theta_{\text{n}}^{\text{i}n}=\theta_{\text{n}}^{\text{o}ut}=0.8$. For all other values of $\theta_{\text{n}}^{\text{i}n}<0.8$, the sheet always swims faster than that in a single fluid, due to the confinement effect from the interfaces.

In the simulations with $\xi =10^{2}$ and $\xi =10^{3}$, the relations between the swimming speed and $\theta_{\text{n}}^{\text{i}n}$ exhibit different trends depending on the value of H. When the fluid interfaces are close to the swimmer (H = 0.16), the swimming speed always decreases with the increase of $\theta_{\text{n}}^{\text{i}n}$. This is illustrated in Figure 14a. The increase of the network volume fraction near the swimmer reduces the viscosity difference between the inner and outer fluid layers. This tends to weaken the confinement effect of the interfaces and reduce the swimming speed. For some values of $\theta_{\text{n}}^{\text{i}n}$, the scaled swimming speed is less than one. For a much larger swimmer-interface distance H = 0.32 (Figure 14b), the swimming speed in the simulations with intermediate frictional coefficient of $\xi =10^{3}$ still goes down as the values of $\theta_{\text{n}}^{\text{i}n}$ increase. The swimming speed 𝒰 is less than ${𝒰}_{0}$ if the value of $\theta_{\text{n}}^{\text{i}n}$ is close to 0.8. By contrast, a non-monotone dependence of the swimming speed on $\theta_{\text{n}}^{\text{i}n}$ is observed for the simulation with $\xi =10^{2}$. In this case, the ratio of $\frac{𝒰}{{𝒰}_{0}}$ is always less than one. With interfaces further away from the swimmer (Figure 14c, H = 0.5), the swimming speed demonstrates non-monotone variations with the increase of $\theta_{\text{n}}^{\text{i}n}$ for both simulations with the intermediate and small values of ξ. In these simulations, the swimmer always moves slower in the mixture than in a single fluid.

## 4. Discussion

In this paper, we carry out a computational investigation of the classical swimming sheet problem within heterogeneous media, based on a two-fluid mixture model and the Immersed Boundary Method. The swimmer moves in a low-viscosity region surrounded by a more viscous bulk fluid, with different fluid layers separated by interfaces. For the situation in which the volume fraction of one fluid vanishes in part of the domain, numerical regularization is used to solve the model equations. The mixture model and numerical regularization obviate the need to track the interface explicitly and enforce the interface conditions. This greatly simplifies the computational algorithm, especially for problems in which the interfaces are dynamic. Good agreement is obtained between numerical simulations and the analytical results for locomotions in layers of single phase fluid. According to our best knowledge, this is the first time that such problems are simulated by a combination of the IB method and the interface-capturing strategy. The numerical results show that both the thrust and the drag on the swimmer increase with the increase viscosity differences between fluid layers and the decrease of the interface-swimmer distance. The rate of increment for the thrust is greater than that for the drag. This always leads to faster swimming, an effect more substantial for swimmers near non-deformable interfaces.

We also investigate locomotions within layers of fluid mixtures. Relative motion and phase separation between components in the mixture can be induced by the waving swimmer. The results illustrate that the swimming speed depends in a non-trivial way on several parameters. With other conditions being the same, larger frictional forces reduce the relative motion and phase separation in the mixture. This always makes the swimmer move faster. For swimming close to the interface, the confinement effect dominates. The reduction of viscosity for the mixture near the swimmer (through the increase of $\theta_{\text{s}}$ or the decrease of $\theta_{\text{n}}$) strengthens the confinement effect from the bulk fluid. This tends to speed up the swimming motion. The situation is more complicated if the interfaces are further away from the swimmer. Depending on the values of H and ξ, the swimming speed can be boosted or reduced by lowering the network fraction near the swimmer. Therefore, swimmers in a two-fluid mixture near free interfaces can move faster or slower than that in a single fluid, depending on their distances from the interface, the composition of the fluid mixture, as well as the magnitude of the frictional force between components in the mixture. This conclusion can be potentially related to the disparity of experimental results on microorganism swimming [28,29].

The computational framework described in the paper can be applied to the study of locomotions of microorganisms within various biofluid media, especially for the cases where swimmers are near free deformable interfaces. The flexibility of the method also makes it a suitable tool to investigate problems in which structural properties of the fluid media are altered by the dynamic swimmer. For example, it is observed that gastric mucin undergoes a phase transition from a viscoelastic gel to a viscous solution, triggered by the urease production from the ulcer-causing pathogen Helicobacter pylori [1]. This localized de-gelling process enables the bacterium to swim through a fluidic pocket (surrounded by gel) and cause infection. The analytical work in [30] based on a simplified model illustrates that the size of the fluid zone plays important roles in determining the physics of motility. However, the gel-sol transition dynamics, chemical reactions, and mucus transport are ignored. Extensions of our computational model can provide a unified framework for the investigation of such locomotive problems in multi-fluid heterogeneous media. The kinetics of rheological changes for gastric mucus may be modeled through the choice of appropriate functional forms for the network stress tensor, which are dependent on the deformation history and chemical concentrations. The process of mucin degradation can be simulated through the addition of reaction terms in equations for $\theta_{\text{n}}$ and $\theta_{\text{s}}$.

## Figures and Tables

**Table 1.** Non-deformable interface (β=0.5): thrust force, drag coefficient, their ratio, and the simulated swimming speed.

kH	Thrust Force ($F_{T}$)	Drag Coefficient (γ)	$-\frac{F_{T}}{\gamma }$	Swimming Speed U
0.16	3.03 × 10^−2^	2.43 × 10^−1^	−1.247 × 10^−1^	−1.262 × 10^−2^
0.25	1.41 × 10^−2^	2.3 × 10^−1^	−6.130 × 10^−2^	−6.151 × 10^−2^
0.4	7.36 × 10^−3^	2.12 × 10^−1^	−3.472 × 10^−2^	−3.479 × 10^−2^

**Table 2.** Deformable interface (β=0.5): thrust force, drag coefficient, their ratio, and the simulated swimming speed.

kH	Thrust Force ($F_{T}$)	Drag Coefficient (γ)	$-\frac{F_{T}}{\gamma }$	Swimming Speed U
0.16	1.23 × 10^−2^	2.43 × 10^−1^	−5.062 × 10^−2^	−5.073 × 10^−2^
0.25	9.54 × 10^−3^	2.3 × 10^−1^	−4.147 × 10^−2^	−4.151 × 10^−2^
0.4	6.63 × 10^−3^	2.12 × 10^−1^	−3.127 × 10^−2^	−3.124 × 10^−2^
